# EXAMINING SOCIAL AND CULTURAL FACTORS AMONG EAST ASIAN FAMILY CAREGIVERS OF PERSONS WITH DEMENTIA

**DOI:** 10.1093/geroni/igad104.1758

**Published:** 2023-12-21

**Authors:** Jessica Cassidy, Kathy Lee, Jihui Lee, Chang Hyun Seo, Alan Kunz Lomelin, Hye-Won Shin, Joshua Grill

**Affiliations:** University of Texas at Arlington, Arlington, Texas, United States; University of Texas at Arlington, Arlington, Texas, United States; University of Pittsburgh, Pittsburgh, Pennsylvania, United States; University of Nevada, Reno, Reno, Nevada, United States; University of Texas at Arlington, Arlington, Texas, United States; University of California Irvine, Irvine, California, United States; University California Irvine, Irvine, California, United States

## Abstract

The purpose of this study was to examine how frequently Korean and Chinese American family caregivers of persons living with dementia utilized home- and community-based formal support services and to assess whether utilization of such services was associated with overall well-being. We collected and analyzed quantitative and qualitative data (N = 62) in effort to understand how cultural and social factors influenced their utilization of home- and community-based services and programs. A total of 36 Korean American and 26 Chinese American family caregivers completed online surveys and individual interviews over the phone. The most frequently used programs by family caregivers of both ethnic groups were in-home care services (33.9%), senior centers (32.3%), family caregiver support programs (24.2%), and transportation services (22.6%). Providing care longer than 108 months (p = 0.047), greater knowledge of information and resources available (p = 0.043) and being a Medicaid recipient (p = 0.008) were associated with utilization of in-home services. Those who utilized nutrition programs and case management were more likely to report higher overall well-being (p < 0.05). Four themes emerged from our qualitative analysis: (1) a lack of knowledge of formal services, (2) language barriers, (3) transportation barriers, and (4) a lack of culturally tailored formal supports. This study emphasizes community-level efforts to increase the utilization of formal support services by developing linguistically and culturally tailored programs, and offering transportation services in existing support programs and online-based education and resources that can be available in different languages.

